# The secondary structure of the TGR5 membrane-proximal C-terminus determines plasma membrane localization and responsiveness towards extracellular ligands

**DOI:** 10.1186/2047-783X-19-S1-S14

**Published:** 2014-06-19

**Authors:** Christoph Gertzen, Lina Spomer, Birte Schmitz, Dieter Häussinger, Verena Keitel, Holger Gohlke

**Affiliations:** 1 Institute for Pharmaceutical and Medicinal Chemistry, Heinrich Heine University, 40225 Düsseldorf, Germany; 2 Clinic of Gastroenterology, Hepatology and Infectious Diseases, Heinrich Heine University, 40225 Düsseldorf, Germany

## 

TGR5 is a structurally unknown bile acid sensing [1] G protein-coupled receptor (GPCR). It is located in different non-parenchymal cells of the liver [2]. Previous studies demonstrated that the deletion of the 35 C-terminal amino acids results in ER retention of the mutated receptor. The membrane proximal C-terminus of GPCRs has been shown to be important for membrane localization of these receptors because it can contain a sorting motif (e.g., the F(X)_6_LL motif). Since the N-terminal part of the C-Terminus of TGR5 contains no known sorting motif, we hypothesize that it must be the secondary structure of the C-terminus that determines TGR5 trafficking [3].

Up to 18 amino acids of the membrane proximal C-terminus of TGR5 wildtype as well as of 8 different substitution and deletion variants within this region were subjected to molecular dynamics (MD) simulations of 600 ns length each using the Amber 11 suite of programs [4]. The starting structure was a straight peptide chain in each case to exclude any bias from prefolded structures. Of the 600 ns of the simulations, the last 500 ns were used for further analyses, respectively. For each variant, the generated conformations were analyzed with respect to the secondary structure sequence by the program “dssp”. All secondary structure sequences of all variants were pooled and hierarchically clustered with the program “R”. Functional analyses of the variants were done using a cAMP reporter gene assay. Membrane localization was determined via FACS analysis.

**Figure 1:**
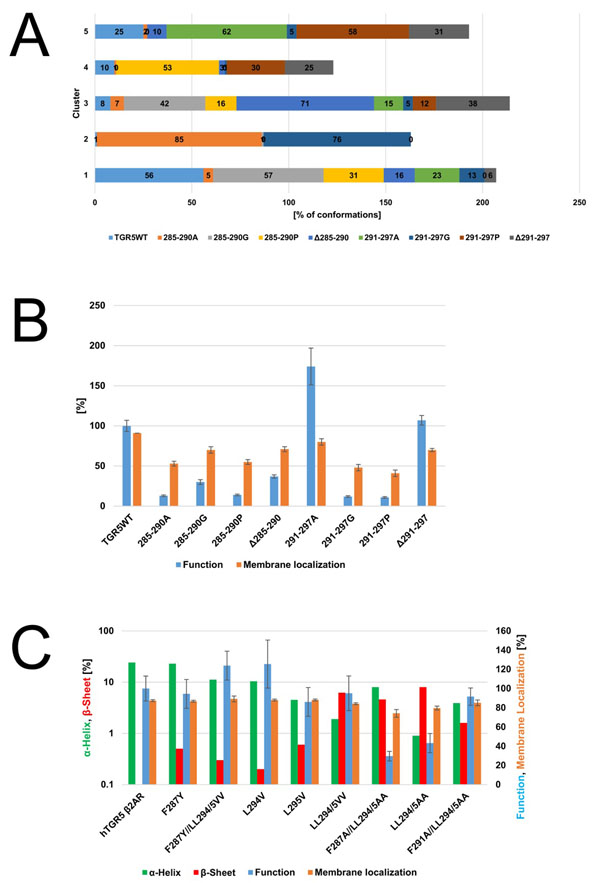
**A** Clustering results of the TGR5 C-terminus WT and mutants according to the secondary structure sequence. The numbers indicate how many percent of the conformations of each variant are found in the respective cluster. **B** Membrane localization and function of the TGR5 C-terminus WT and mutants. The membrane localization was determined by FACS assay; the function was determined by cAMP reporter gene assay (n > 8). **C** Secondary structure content, membrane localization, and function of the TGR5β_2_AR chimera variants. The secondary structure content was averaged over all residues and over the simulation time; the membrane localization was determined by FACS assay; the function was determined by a cAMP reporter gene assay (n > 8). Panels adapted from ref. [3].

The clustering shows that clusters 1 and 5 are mainly comprised of the wildtype (WT) and variants 285-290G, 291-279A, and Δ291-297 (Figure 1A). These variants show intermediate to high functionality up to 174% of the WT and a membrane localization of more than 70% (Figure 1B). The conformations in these clusters show a predominant α-helix formation. Cluster 2 contains the variants 285-290A and 291-297G (Figure 1A). These variants show a functionality < 13% of the WT and a membrane localization < 53% (Figure 1B). The most frequently occurring secondary structure in cluster 2 is a β-sheet. Finally, clusters 3 and 4 contain the variants 285-290P, Δ285-290, and 291-297P. These variants show a low to intermediate functionality (11% to 37% of the WT) and membrane localization (41% to 77%) (Figure 1B). The most frequently occurring secondary structure in clusters 3 and 4 is a loop formation. These results show that an α-helix formation in the variants C-terminus is associated with a high functionality and membrane localization, as can be seen for clusters 1 and 5. In contrast, β-sheet or loop formation is associated with reduced functionality and membrane localization, as can be seen for clusters 2 to 4. Overall, this suggests that mere secondary structure content rather than a specific amino acid sequence of the membrane-proximal part of the C-terminus determine PM localization and function of TGR5.

As a proof of concept, we constructed a chimera of TGR5 with the 13 membrane-proximal amino acids of the C-terminus replaced by respective amino acids of the C-terminus of the β_2_-adrenergic receptor (β_2_AR). These β_2_AR residues form an α-helix in the β_2_AR crystal structure and show no sequence identity to the exchanged amino acids from TGR5. As expected, this chimera was correctly sorted to the plasma membrane and showed a similar functional activity as the TGR5 wildtype. As the constructed chimera does contain a F(X)_6_LL sorting motif within the β_2_AR part, however, we also investigated the influence of the sorting motif by mutation studies and subsequent experimental and computational characterization of the variants. The TGR5β_2_AR chimera and its F287Y variant both show a membrane localization of approx. 87%, and the mutant a functionality of > 95% of the chimera WT (Figure 1C). Both variants also show the highest α-helix formation, averaged over all residues, with > 23% over the course of the MD simulations but no β-sheet formation (Figure 1C). Variants F287Y//LL294/5VV, L294V, and L295V show functionalities between 86% and 123% of the chimera WT and membrane localizations of approx. 88% (Figure 1C). While the α-helix content of these variants is reduced to 5% to 11%, their β-sheet content is still < 0.6% (Figure 1C). In contrast, variant LL294/5VV shows a β-sheet content of 6% but only an α-helicality of 2%, which is accompanied by a reduced membrane localization of 84% (Figure 1C). Along these lines, the variants F287A//LL294/5AA and LL294/5AA show a low functionality between 30% and 43% of the chimera WT and a reduced membrane localization of < 79%. Their respective α-helicality is 8% and 0.9%, but their respective β-sheet content is as high as 4.6% and 8%. Taken together, the TGR5β_2_AR variants reveal a reduced functionality and membrane localization if the α-helicality decreases and the β-sheet content increases in the membrane-proximal part of their C-termini. These results are consistent with our observations on the TGR5 variants and further strengthen our hypothesis that an α-helix formation in that region fosters membrane localization of TGR5.

For a final validation, we created the F291A mutant of the LL294/5AA variant of TGR5β_2_AR. This was done because our MD simulations had identified a strong hydrophobic contact between F291 and A295 in the LL294/5AA variant, leading to a β-sheet formation between these two residues for 60% of the simulated time. In contrast, MD simulations predicted that this contact is broken in the triple alanine mutant F291A//LL294/5AA, consequently restoring α-helicality (to 3.9%) and reducing the β-sheet content (to 1.6%) (Figure 1C). Experimental testing confirmed our expectation in that the triple alanine mutant shows an increased membrane localization (85.1%) and functionality (91.6% of the chimera WT) (Figure 1C). Thus, we were able to rescue a variant with impaired functionality and membrane localization by introducing another mutation that had been predicted to restore α-helicality in the membrane-proximal part of their C-termini.

In summary, we first demonstrated that an α-helix in the membrane proximal C-terminus of TGR5 fosters membrane localization of the receptor while β-sheet or loop formation in this region leads to ER retention and a loss of function. Second, a chimera with a known α-helical C-terminus of β_2_AR showed a membrane localization and function similar to the TGR5 WT. Additionally, variants of the TGR5β_2_AR chimera with mutations in the β_2_AR sorting motif showed a likewise relationship between secondary structure content and membrane localization and function as observed for the variants of the TGR5 WT. Finally, we demonstrated that a non-functional chimera variant can be rescued by introducing an additional mutation that restores α-helicality. We thus conclude that the secondary structure of the TGR5 membrane-proximal C-terminus, which forms an α-helix according to our MD simulations, is the determining factor for plasma membrane localization and responsiveness towards extracellular ligands.
